# Trapped: Extraction of an implantable cardioverter-defibrillator lead victim to percutaneous interventional left ventricular volume reduction

**DOI:** 10.1016/j.hrcr.2024.07.011

**Published:** 2024-07-15

**Authors:** Benito Baldauf, Marzia Giaccardi, Hendrik Bonnemeier

**Affiliations:** ∗ Christian-Albrechts University Kiel, Kiel, Germany; † Institute of Life Science, Hochschule Bremerhaven, Bremerhaven, Germany; ‡ Ospedale Santa Maria Annunziata, Firenze, Italy

**Keywords:** Transcatheter left ventricular reduction, Implantable cardioverter-defibrillator, Lead extraction, Cardiac implantable electronic device, Complication, Severe adverse event

**Key Teaching Points**

• The formation of a ventricular aneurysm, resulting in reduced contractility and arrhythmias, highlights the substantial impact of myocardial scar tissue on cardiac function, underscoring the need for customized interventions for younger individuals with severe postinfarction myocardial damage we increasingly encounter during everyday routine.
• Our patient’s response to the hybrid approach and subsequent improvement in left ventricular ejection fraction to nearly normal levels (48%) after surgery reflects the dynamic nature of heart failure management, where advanced surgical techniques can significantly impact outcomes.
• The accidental entrapment of the right ventricular lead and subsequent revision procedure highlight the importance of vigilant implantable cardioverter-defibrillator (ICD) system interrogation and management postintervention with increasingly complex medical devices.
• Modern medical devices for lead extraction in cardiovascular implantable electronic device complications offer enhanced precision, reduced procedural risks, and improved patient outcomes through minimally invasive techniques employing specialized extraction tools, even in the most complex of cases.
• This case calls into question the necessity of reimplanting transvenous ICDs in patients with improved cardiac function postsurgery. It highlights the evolving landscape of heart failure management, where contemporary medical therapy may reduce the need for certain interventions, warranting further research into the criteria for ICD placement.

## Introduction

The management of cardiovascular implantable electronic devices (CIEDs) and complex interventions like minimally invasive left ventricular (LV) reduction presents significant challenges and potential adverse events. CIEDs, such as pacemakers and implantable cardioverter-defibrillators (ICDs), are vital for managing arrhythmias and preventing sudden cardiac death (SCD) but can lead to complications, including infections, lead dislodgement, and mechanical failures.

Minimally invasive LV reduction procedures, aimed at improving cardiac function in heart failure patients, also carry risks such as residual shunts, arrhythmias, and damage to cardiac structures. The combination of surgical and percutaneous techniques in these procedures adds to the complexity.

This case study of a 38-year-old patient undergoing LV reduction and subsequent ICD revision illustrates the delicate balance between therapeutic benefits and potential risks of modern-era interventions.

## Case report

A 38-year-old White woman presented to the emergency department complaining of dyspnea on exertion and paroxysmal nocturnal dyspnea. She reported a history of continued smoking, equivalent to 15 pack-years, and a past medical history significant for percutaneous coronary intervention with placement of a drug-eluting stent in the proximal anterior left descending artery a decade ago, following an acute coronary thrombosis. Additionally, 6 years prior, she underwent implantation of a dual-chamber ICD for primary preventative intention,^1^ with a left ventricular ejection fraction (LVEF) of 30%. Her current medication regimen included an angiotensin-converting enzyme inhibitor, 2 diuretics, oral antidiabetics, and platelet inhibition.

Further investigation, including laboratory tests and cardiac ultrasound studies, was initiated to rule out acute myocardial ischemia and pulmonary embolism. Echocardiography revealed a reduced LVEF of 28%, attributed to the development of an LV aneurysm causing apicoseptal akinesia in a globally enlarged heart. Coronary angiography showed no progression of coronary vessel disease, and ICD interrogation indicated normal threshold values. Computed tomography confirmed normal pulmonary vessel anatomy and no pulmonary embolism.

The patient’s reduced cardiac function potentially stemmed from scar tissue formation in the heart muscle following myocardial infarction, resulting in decreased contractility, myocardial fibrosis, and the development of a ventricular aneurysm. In addition to decreased cardiac output and arrhythmias, ventricular aneurysms increase the risk of thrombus formation and subsequent complications.

**Figure 1:**
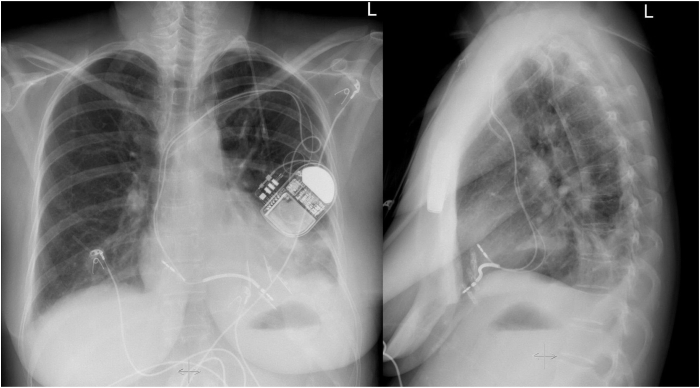
Postprocedural radiograph.

The heart team deliberated on the optimal management approach, considering minimal invasive cardiac reconstruction to mechanically “isolate” the scar tissue from the functioning myocardium vs ventricular aneurysm removal during open chest surgical procedure. The patient opted for the hybrid approach with off-pump LV reconstruction.^2^ During the procedure, 2 pairs of external and internal anchors were successfully placed to isolate or compress the aneurysm, aligning the contractile parts of the LV walls (Figure 1). The internal anchoring device is positioned anteroseptally at the right ventricular (RV) apex using a catheter, deploying a titanium anchor coated with polyester and a tether wire. The tether wire is then retrieved via a trocar introduced through a thoracotomy across the LV wall and septum in an apical position. Subsequently, the external anchoring device is inserted through the same thoracotomy over the tether wire to fold the akinetic ventricular tissue by retracting the wire.

**Figure 2:**
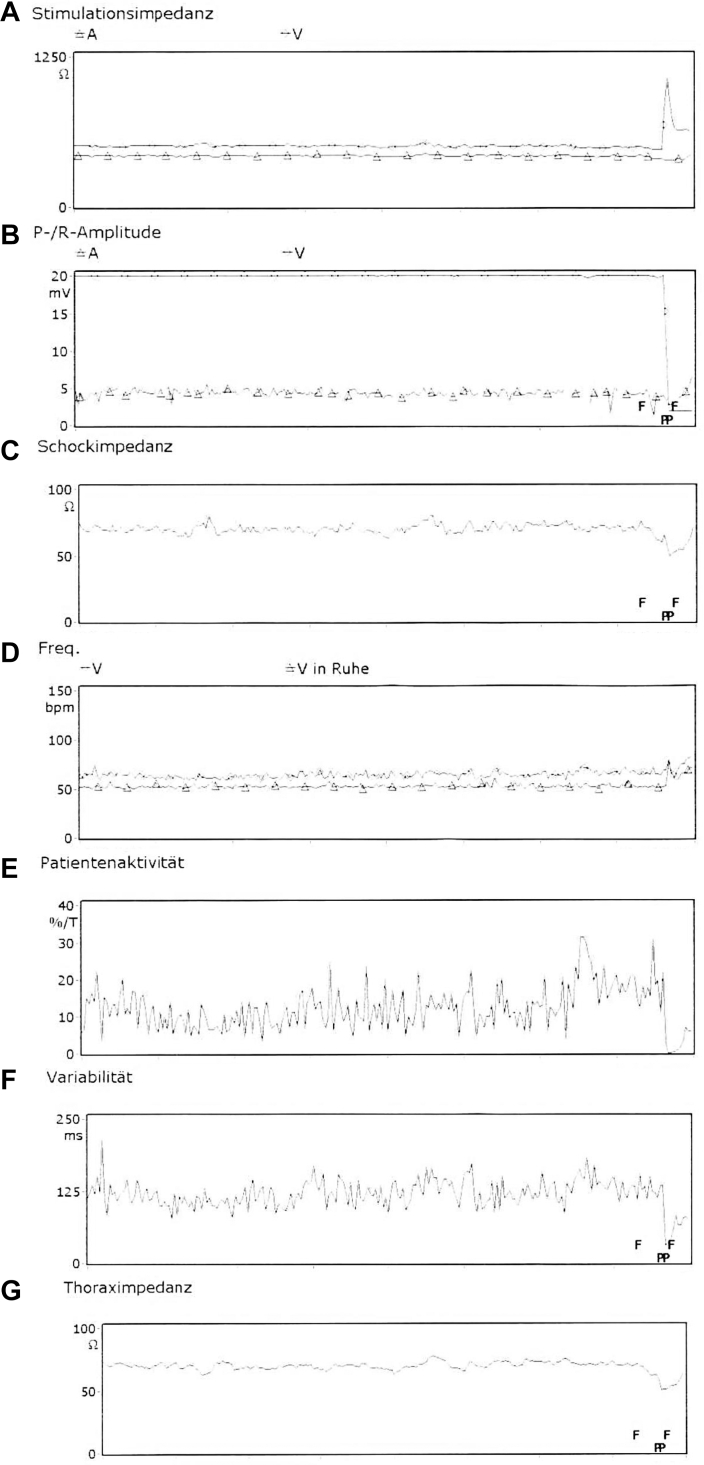
Implantable cardioverter-defibrillator interrogation showing a marked sharp increase in lead impedance (**A**) and heart rate (**D**), a reduction in P/R wave amplitude (**B**), shock impedance (**C**), thorax impedance (**G**), variability (**F**), and patient activity (**E**) at the time of the intervention.

**Figure 3:**
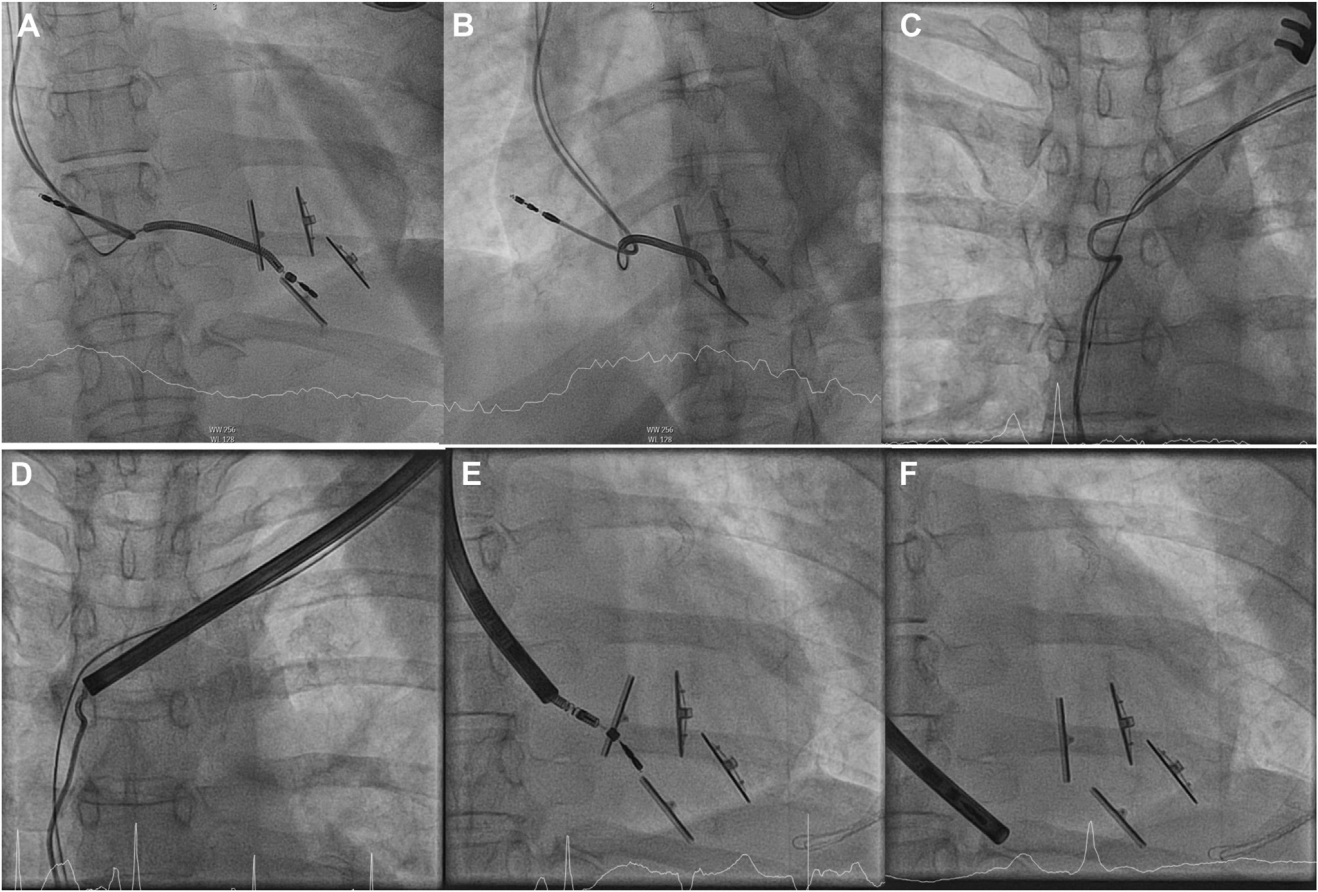
**A, B:** Images illustrating the accidental entrapment of the right ventricular (RV) lead between the RV anchors. **C–F:** Periprocedural images during atrial (**C, D**) and RV lead (**E, F**) extraction.

Despite improvements in cardiac function postprocedure, dysfunction of the RV lead was noted during ICD system interrogation (Figure 2; note the sharp rise in pacing impedance [Figure 2A] and heart rate [Figure 2D], the drop in P/R amplitude [Figure 2B], shock [Figure 2C], thorax impedance [Figure 2G], variability [Figure 2F], and patient activity [Figure 2E] at the time of the intervention). Imaging revealed accidental entrapment of the RV lead between the RV anchors (Figure 3A and 3B). This unexpected complication necessitated a revision of the ICD system. Intraprocedural fluoroscopy confirmed entrapment (Figure 3A and 3B), and a surgical procedure, including transvenous extraction of all hardware and removal of fibrotic tissues, was performed (Figure 3C–3F).

Recovery was uneventful, and subsequent cardiac ultrasound study revealed a close-to-normal LVEF of 48%. Given the lack of intervention by the ICD during its dwell time, reimplantation of a transvenous ICD was deferred (Supplemental Figure 1).

## Discussion

While the hybrid approach presents a unique minimally invasive option, its efficacy compared to guideline-based medical therapy or open chest surgery remains uncertain. Complications, including residual shunts, induction of arrhythmia, or the unlikely event of accidental entrapment of the RV lead in this case, underscore the potential risks associated with this procedure and necessitate careful postprocedural monitoring. Additionally, this case highlights the evolving landscape of heart failure management, where contemporary medication changes risks for adequate therapy from ICDs in preventing SCD, prompts further contemporary research to elucidate ICD placement necessity for preventing SCD.^3^

During the placement of the anchors, cranial kinking of the shock lead was observed proximal to the lead tip. However, even at the most extreme angles, initial evaluation ruled out lead tip dislocation. This case is among the first performed using a minimally invasive hybrid approach globally, and no similar cases have been documented. The adverse event observed is likely attributable to a more physiological downward positioning of the apex postalignment, compared to its previous leftward deviation. This incident prompted the adoption to potentially exclude patients from minimally invasive transcatheter LV reduction therapy, in case indwelling CIED leads are present.

The increasing complexity of procedures underscores the importance of careful planning and follow-up care. This case emphasizes the importance of exercising caution when implementing novel interventions to safeguard patient well-being.

## Funding Sources

Supported by the DEAL consortium agreement between the German Rectors Conference and Elsevier.

## Disclosures

Benito Baldauf: Medical consultant/part of the advisory board: Abbott, Bioline Supply, Biotronik, Cablon NL, CRM Microport, Crosstec GmbH, Drugsales Limited, Kappamed, Kimal PLC, M3 Medical/Ecclipse Medical, Medival SRL, Medtronic, Philips/Spectranetics, Sintec SRL, Tauro-Implant GmbH, Tauropharm GmbH, Transcutan. The other authors have nothing to declare.

## Appendix Supplementary Data

Supplemental Figure 1 post-extraction X-ray (in bed)
