# Supplementary figures and images for: Trapped: Extraction of an implantable cardioverter-defibrillator lead victim to percutaneous interventional left ventricular volume reduction
Source: HeartRhythm Case Rep. 2024 Jul 15;10(10):734–7. doi: 10.1016/j.hrcr.2024.07.011 (PMC11628816; doi:10.1016/j.hrcr.2024.07.011)

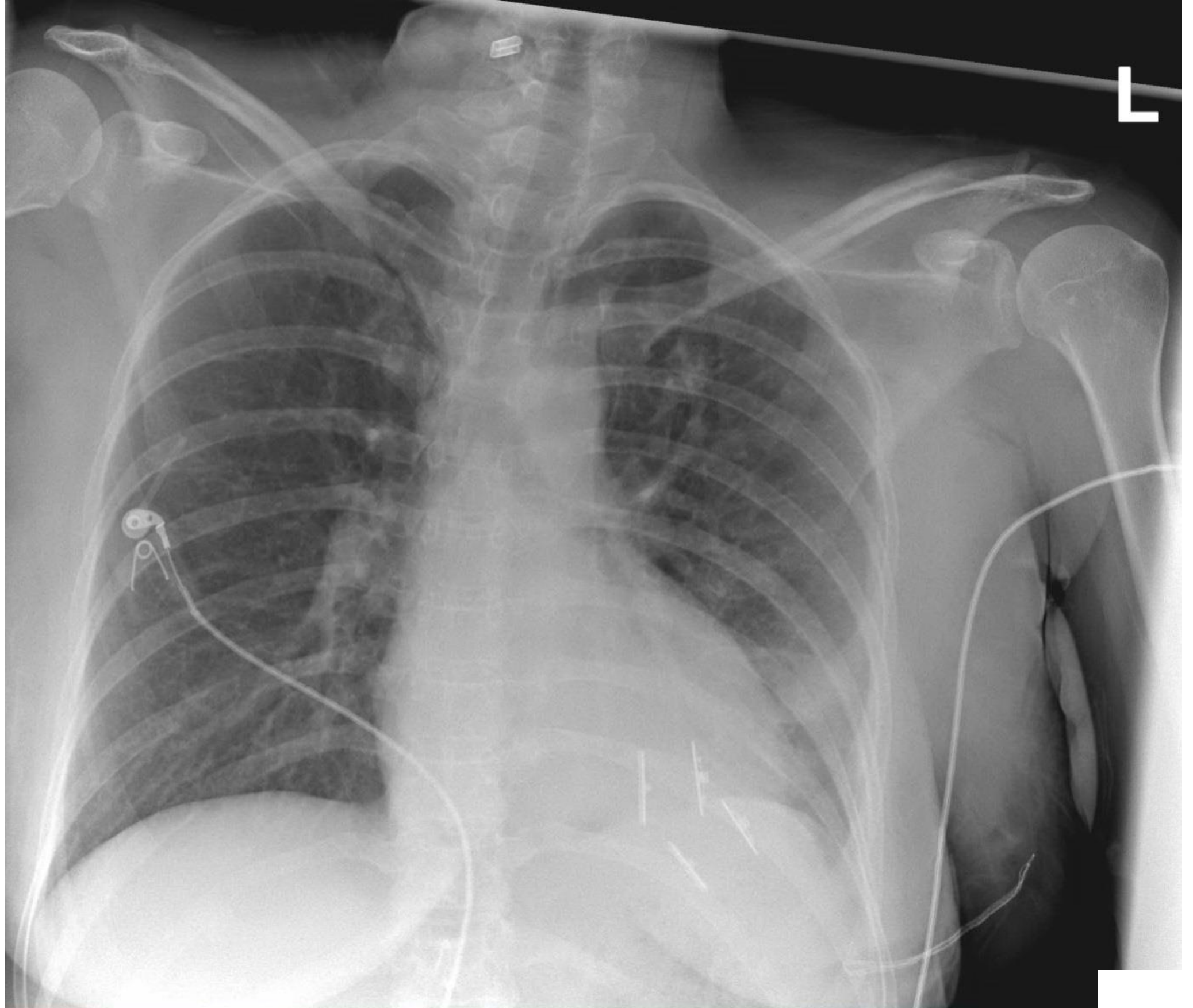

Supplement: Supplemental Figure 1 — post-extraction X-ray (in bed) [file mmc1.pdf]
